# Selectively recommend ^18^F-FDG PET/CT for patients with de novo nasopharyngeal carcinoma in endemic areas

**DOI:** 10.1186/s13014-021-01954-8

**Published:** 2021-11-27

**Authors:** Chuanben Chen, Ting Xu, Xiufang Qiu, Shihan Xie, Ziqing You, Yixin Hu, Yinghong Zheng, Zewei Liang, Chaoxiong Huang, Taojun Chen, Li Li, Jing Liu, Zhaodong Fei

**Affiliations:** 1 Fujian Medical University Cancer Hospital, Fujian Cancer Hospital, Fujian Medical University, Fuzhou, Fujian People’s Republic of China; 2 Fujian Medical University, Fuzhou, Fujian People’s Republic of China; 3 Department of Radiation Oncology, Fujian Medical University Cancer Hospital, Fujian Cancer Hospital, Fujian Medical University, Fuma Road, Fuzhou, 350014 Fujian People’s Republic of China

**Keywords:** Nasopharyngeal carcinoma, [^18^F] Fluorodeoxyglucose positron emission tomography, Metastasis, Economic evaluation, Cost-effectiveness analysis

## Abstract

**Introduction:**

To identify the subset of patients with de novo nasopharyngeal carcinoma (NPC) for whom [^18^F] fluorodeoxyglucose positron emission tomography and computed tomography (^18^F-FDG PET/CT) should be recommended, and to determine whether PET/CT is a cost-effective decision for precise M staging in endemic areas.

**Materials and methods:**

Retrospective analysis of data of 4469 patients diagnosed with de novo NPC between January 2014 and December 2019. The detection rate of distant metastasis was compared between different groups. Univariate and multiple logistic regression analysis was applied to identify the risk factors for distant metastasis. The cost-effectiveness of the diagnostic strategies was assessed.

**Results:**

The detection rate of distant metastasis in the whole cohort was 5.46%. In multivariate analysis, male sex, T3-4 stage, N2-3 stage, and high plasma Epstein-Barr virus (EBV) DNA (≥ 14,650 copies/mL) were risk factors for distant metastases. NPC patients with T3-4 stage combined with N2-3 stage, high EBV DNA combined with male sex, or N2-3 stage combined with high EBV DNA were defined as recommended group with relatively higher tendency for metastasis. Distant metastasis incidence in recommended group and unrecommended group were 10.25% and 1.75%, respectively (*P* < 0.001). In the recommended group, PET/CT significantly improved the detection rate of distant metastasis (13.25% vs 9.02%, *P* = 0.005). Cost-effectiveness analysis revealed that additional cost for every one percent increase in distant metastasis detection rate was $22,785.58 in the recommended group (< Willingness-to-pay (WTP) threshold of $32,700.00) and $310,912.90 in the unrecommended group.

**Conclusions:**

In patients with de novo NPC, the tendency for metastasis can be predicted based on clinical parameters. ^18^F-FDG PET/CT should be selectively recommended for the subset of patients with a relatively higher tendency for metastasis.

## Introduction

Nasopharyngeal carcinoma (NPC) is a highly aggressive malignant tumor prevalent in South China and Southeast Asia [1]. According to the Global Cancer Statistics, an estimated 133,000 new cases of NPC were diagnosed in 2020 worldwide [2]. Approximately 4–10% patients with NPC have distant metastasis at the time of diagnosis [3–7]. NPC commonly metastasizes to bone, lung, and liver. However, the difficulty of obtaining pathological biopsy limits the diagnostic accuracy for distant metastasis. Conventional work-up (CWU) with chest computed tomography (CT), abdominal ultrasonography, and skeletal scintigraphy are widely used to detect metastasis in patients with NPC because of their low cost and wider accessibility [8]. Nevertheless, ultrasonography is relatively operator-dependent and has poor sensitivity for differentiating between benign and metastatic tumors. Skeletal scintigraphy seems to have low sensitivity for detection of early bone or bone marrow metastasis [9].

In recent years, [^18^F] fluorodeoxyglucose positron emission tomography and computed tomography (^18^F-FDG PET/CT) has become popular due to its unique capability to image metabolically active lesions. The combination of a dedicated PET scanner and helical CT enables high-resolution morphologic imaging and integrated functional imaging. Conventional imaging modalities such as ultrasound and CT allow detection of lesions based on morphologic alterations, whereas PET enables quantitative assessment of biochemical, physiological, and metabolic alterations in vivo, and these alterations usually precede the anatomical changes [10, 11]. Many studies have demonstrated excellent sensitivity and specificity of PET/CT for N and M classification in newly diagnosed NPC and for the diagnosis of residual or recurrent NPC [9, 12–14].

However, the high cost of ^18^F-FDG PET/CT precludes its use in a vast majority of NPC patients, especially in China and other endemic areas in southeast Asia, which account for > 70% new cases annually. Given the current health care economic environment, the aim of the present study was to identify the risk factors for distant metastasis in a large cohort of real-world patients with newly diagnosed NPC and to selectively recommend ^18^F-FDG PET/CT to de novo NPC patients with relatively higher tendency for metastasis.

## Material and methods

### Patients

This retrospective analysis was approved by the ethics committee at our center (No. YKT2020-O11-01). A total of 4469 newly diagnosed NPC patients with a pathological diagnosis at our center between January 2014 and December 2019 were enrolled in the study. The T and N stage was reclassified according to the 8th edition of the American Joint Committee on Cancer (AJCC) staging system.

#### ^18^F-FDG PET/CT for distant metastasis

Owing to the high cost of PET/CT, its use depended on the patient’s willingness, affordability, and the physician’s suggestion. Out of 4469 patients, 1171 patients opted to undergo PET/CT for detection of distant metastases. The specific methods were detailed in our previous study [15].

#### Conventional work-up for distant metastasis

A total of 3298 patients in our cohort underwent CWU for M classification. CWU consisted of chest CT, abdominal ultrasonography, and whole-body skeletal scintigraphy.

#### Detection of distant metastasis

Patients with suspicious distant metastases lesions were subjected to additional examinations, including CT, magnetic resonance imaging (MRI), and/or biopsy. The clinical diagnosis of distant metastases was confirmed by at least two image examinations if a biopsy of the suspicious tissue was not available [16, 17]. If additional examinations yielded negative results or were not feasible, suspicious lesions were not considered as distant metastases.

### Evaluation of plasma Epstein-Barr virus (EBV) DNA

Plasma EBV DNA was routinely measured by quantitative polymerase chain reaction (q-PCR) before treatment. Receiver operating characteristic (ROC) curve analyses were performed to evaluate the ability of plasma EBV DNA to predict distant metastases by choosing the optimal cut-off value that showed the best trade-off between sensitivity and specificity.

### Economic evaluation

For the assessment of economic benefit, we performed a cost-effectiveness analysis from the perspective of traditional payers. In this study, direct costs of PET/CT and CWU for primary staging were calculated as total costs, irrespective of the kind of payment (insurance or no insurance). The cost of plasma EBV-DNA and additional evaluations for clarifying a positive test or investigations performed during the follow-up were not included in the economic analysis. For PET/CT and CWU, the charges notified by the Medical Insurance Administration Bureau of Fujian, China in 2019 were used. The detection rates for distant metastases in de novo NPC by PET/CT and CWU were considered as measures of effectiveness. The reference of cost-effectiveness analysis was incremental cost-effectiveness ratio (ICER). The ICER was calculated by dividing the total cost difference between the PET/CT group and the CWU group by the difference in effectiveness between the two groups. All costs are expressed in U.S. Dollars (USD) based on the currency exchange rate in December 2019 [1 USD equals 6.98 China Yuan (CNY)]. Discounting was not applied for detection rates as the staging evaluation were assumed to occur within a single time period. Willingness-to-pay (WTP) is the predetermined threshold employed in economic evaluations which helps determine the greatest amount that should be paid for every additional unit of effectiveness gained from a new intervention [18]. Cost-effectiveness acceptability analysis was conducted to evaluate optimal strategies at given WTP thresholds [19, 20]. The WTP threshold was set as three times the Gross Domestic Product (GDP) per capita in 2019, which was $32,700.00 in China.

### Statistical analysis

Age, sex, plasma EBV DNA level, T stage, and N stage were analyzed to identify the risk factors for distant metastasis. The optimal cut-off value of EBV DNA level was determined using ROC curve analysis. The rates were compared using the Chi-squared test. Univariate and multiple logistic regression analyses were applied to analyze the association between the risk factors and distant metastasis; the odds ratios (ORs) and 95% confidence intervals (CIs) were calculated to identify the major risk factors. All statistical analyses were performed using the SPSS 22.0 (IBM Corporation, Chicago, IL, USA) and GraphPad Prism 7.0. Two-sided *P* values < 0.05 were considered indicative of statistical significance.

## Results

### Patient characteristics

The baseline demographic and clinical characteristics of the study population are presented in Table 1. The median age of patients was 49 years (range 5–87), and the male-to-female ratio was 2.72:1.

**Table 1:** Baseline characteristics of 4469 NPC patients at primary diagnosis

Characteristic	Total N = 4469 (%)	PET/CT group N = 1171 (%)	CWU group N = 3298 (%)
Gender			
Male	3269 (73.15)	871 (74.38)	2398 (72.71)
Female	1200 (26.85)	300 (25.62)	900 (27.29)
Age			
≥ 50	2122 (47.48)	535 (45.69)	1587 (48.12)
< 50	2347 (52.52)	636 (54.31)	1711 (51.88)
T stage			
T1	820 (18.35)	227 (19.39)	593 (17.98)
T2	793 (17.74)	212 (18.10)	581 (17.62)
T3	1627 (36.41)	468 (39.97)	1159 (35.14)
T4	1229 (27.50)	264 (22.54)	965 (29.26)
N stage			
N0	375 (8.39)	95 (8.11)	280 (8.49)
N1	1593 (35.65)	351 (29.97)	1242 (37.66)
N2	1569 (35.11)	425 (36.29)	1144 (34.69)
N3	932 (20.85)	300 (25.62)	632 (19.16)
M stage			
M0	4225 (94.54)	1084 (92.57)	3141 (95.24)
M1	244 (5.46)	87 (7.43)	157 (4.76)
Plasma EBV DNA			
Low*	3553 (79.50)	859 (73.36)	2694 (81.69)
High**	916 (20.50)	312 (26.64)	604 (18.31)

EBV, Epstein-Barr virus

*Low EBV DNA level of < 14,650 copies/ml

**High EBV DNA level of ≥ 14,650 copies/ml

### Detection of metastasis by PET/CT and CWU

The detection rate of distant metastasis in the whole cohort was 5.46%. The rate of detection of distant metastases in PET/CT group was 7.43%, and in CWU group was 4.76%.

### EBV DNA cut-off level for predicting distant metastasis

ROC curve analysis was used to determine the optimal pre-treatment cut-off plasma EBV DNA level for predicting distant metastasis. The optimal cut-off EBV DNA level was 14,650 copies/mL (area under curve [AUC] = 0.708, sensitivity = 0.513, specificity = 0.811). We observed a significant difference in distant metastasis rates between patients with high EBV DNA level (≥ 14,650 copies/mL) and low EBV DNA levels (< 14,650 copies/mL). Distant metastasis rate was significantly higher in high EBV DNA group compared to low EBV DNA group (12.77% vs. 3.57%, *P* < 0.001).

### Risk factors for distant metastasis

**Fig. 1:**
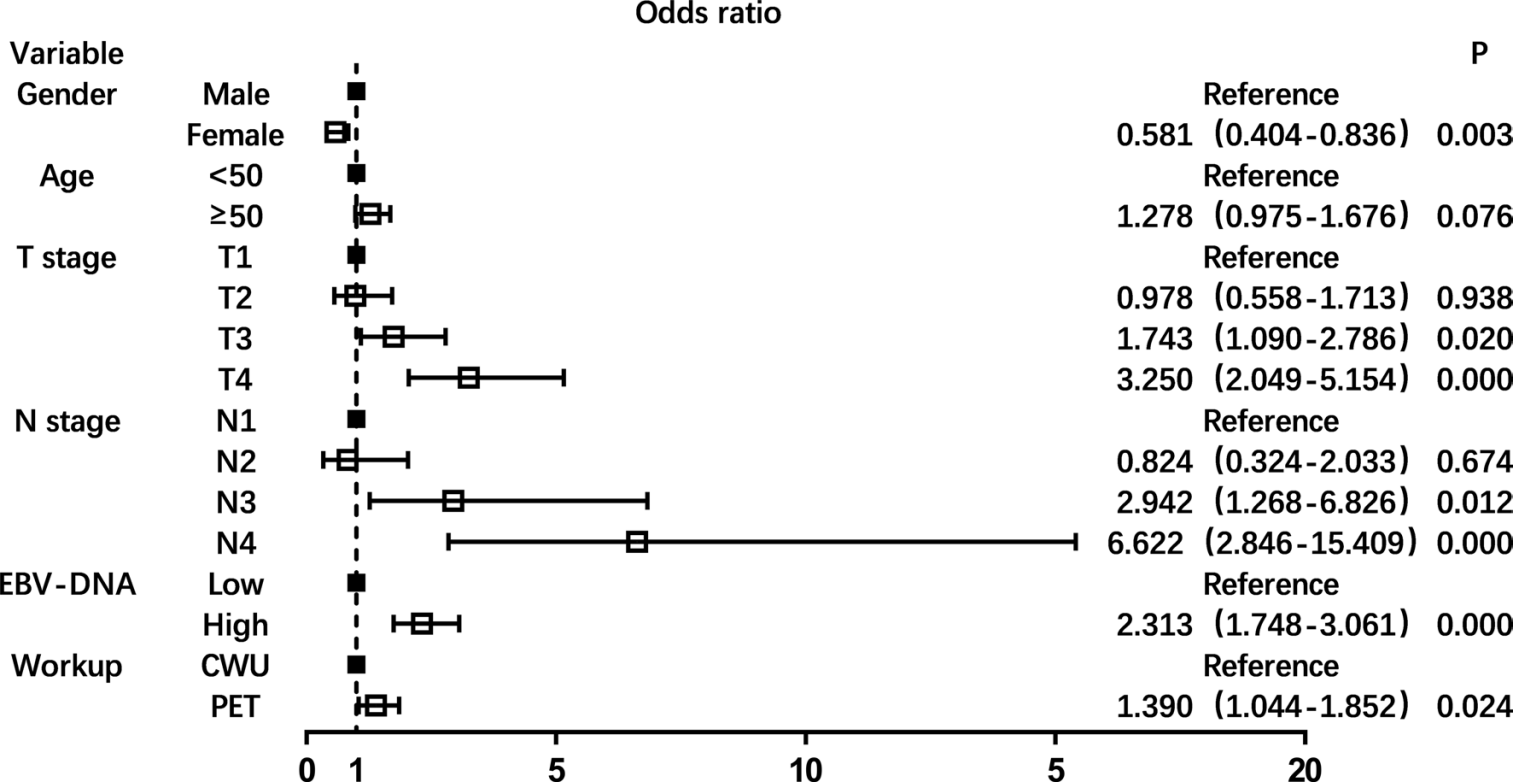
Multiple logistic regression analysis of the risk factors related to distant metastasis in the whole cohort of de novo NPC patients

Univariate and multiple logistic regression analyses were performed to analyze the risk factors related to distant metastasis. The results of univariate analysis are displayed in Table 2. Sex, T stage, N stage, plasma EBV DNA level, and detection method showed a significant correlation with distant metastasis (*P* < 0.05 for all). The incidence of distant metastasis increased with increasing T and N stage (*P* < 0.001 for both) and distant metastasis rate was much higher in T4 and N3 patients (8.95% and 12.77%, respectively), as well as in T3-4 and N2-3 patients (6.69% and 8.52%, respectively). In multivariate analysis, female sex (OR = 0.581, 95% CI 0.404–0.836), T3 stage (OR = 1.743, 95% CI 1.090–2.786), T4 stage (OR = 3.250, 95% CI 2.049–5.154), N2 stage (OR = 2.942, 95% CI 1.268–6.826), N3 stage (OR = 6.622, 95% CI 2.846–15.409), and high plasma EBV DNA (OR = 2.313, 95% CI 1.748–3.061) were independent predictors of distant metastasis (*P* < 0.05 for all) (Fig. 1). Specifically, male sex, T3-4 stage, N2-3 stage, and high plasma EBV DNA level were identified as risk factors for distant metastases in patients with de novo NPC.

**Table 2:** Univariate analysis of the risk factors related to distant metastasis in de novo NPC patients

	DM positive N = 244(%)	DM negative N = 4225(%)	OR (95%CI)	*P*
Gender				0.000
Male	206 (84.43)	3063 (72.50)	1	–
Female	38 (15.57)	1162 (27.50)	0.486 (0.342–0.692)	–
Age				0.062
≤ 50	130 (53.28)	1992 (47.15)	1	–
> 50	114 (46.72)	2233 (52.85)	1.278 (0.987–1.656)	–
T stage				0.000
T1	25 (10.25)	795 (18.82)	1	0.000
T2	28 (11.48)	765 (18.11)	1.164 (0.673–2.014)	0.587
T3	81 (33.20)	1546 (36.59)	1.666 (1.055–2.630)	0.028
T4	110 (45.08)	1119 (26.48)	3.126 (2.006–4.872)	0.000
N stage				0.000
N0	6 (2.46)	369 (8.73)	1	0.000
N1	25 (10.25)	1568 (37.11)	0.981 (0.399–2.407)	0.966
N2	94 (38.52)	1475 (34.91)	3.919 (1.704–0.017)	0.001
N3	119 (48.77)	813 (19.24)	9.002 (3.928–20.629)	0.000
Plasma EBV DNA				0.000
Low*	127 (52.05)	3426 (81.09)	1	–
High**	117 (47.95)	799 (18.91)	3.950 (3.038–5.137)	–
Group				0.001
CWU	157 (64.34)	3141 (74.34)	1	–
PET	87 (35.66)	1084 (25.66)	1.606 (1.225–2.1.5)	–

DM, distant metastasis

*Low EBV DNA level of < 14,650 copies/ml

**High EBV DNA level of ≥ 14,650 copies/ml

### Risk of distant metastasis

**Fig. 2:**
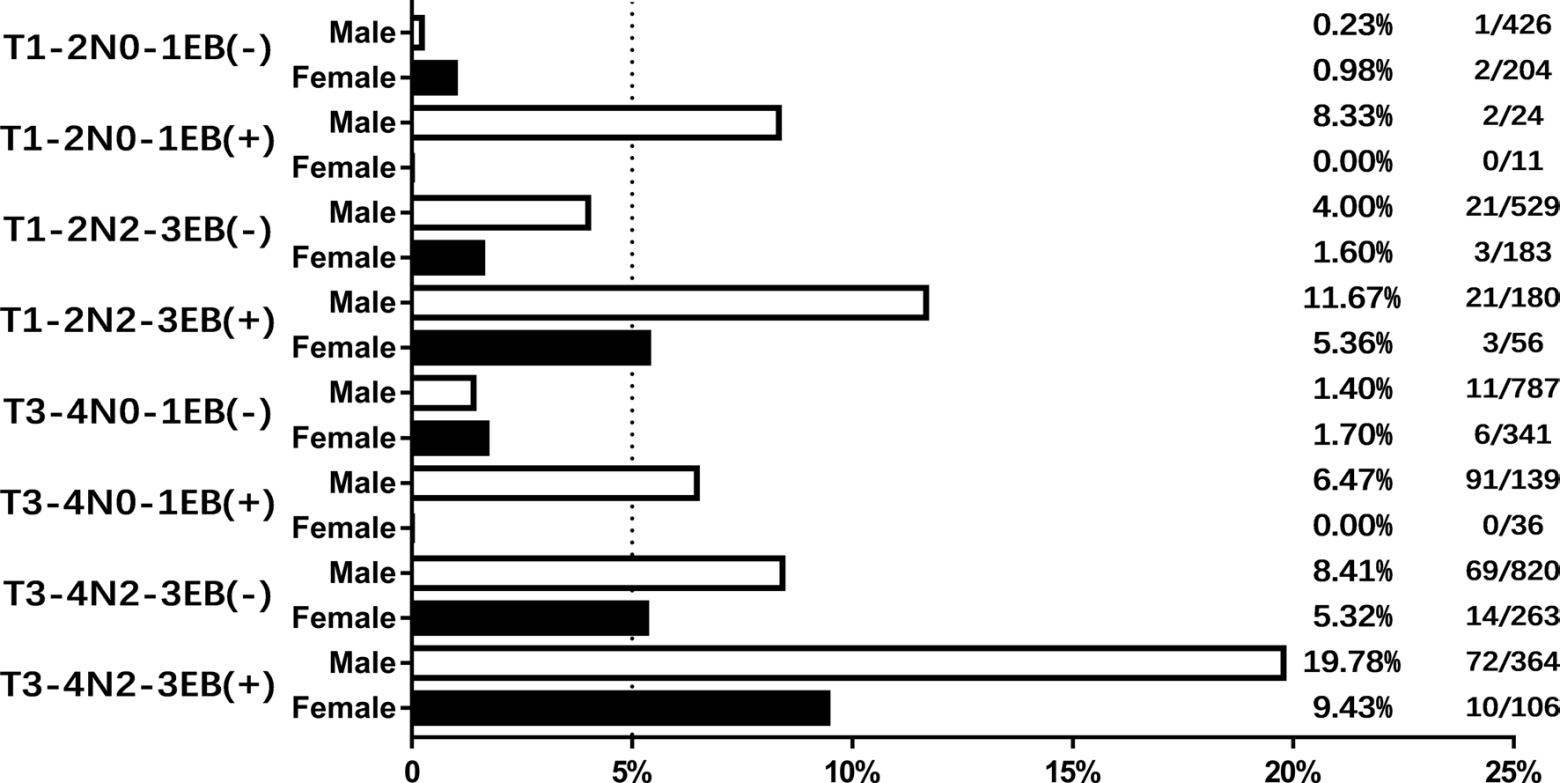
16 subgroup situations by the combinations of 4 risk factors and its distant metastasis rates

**Fig. 3:**
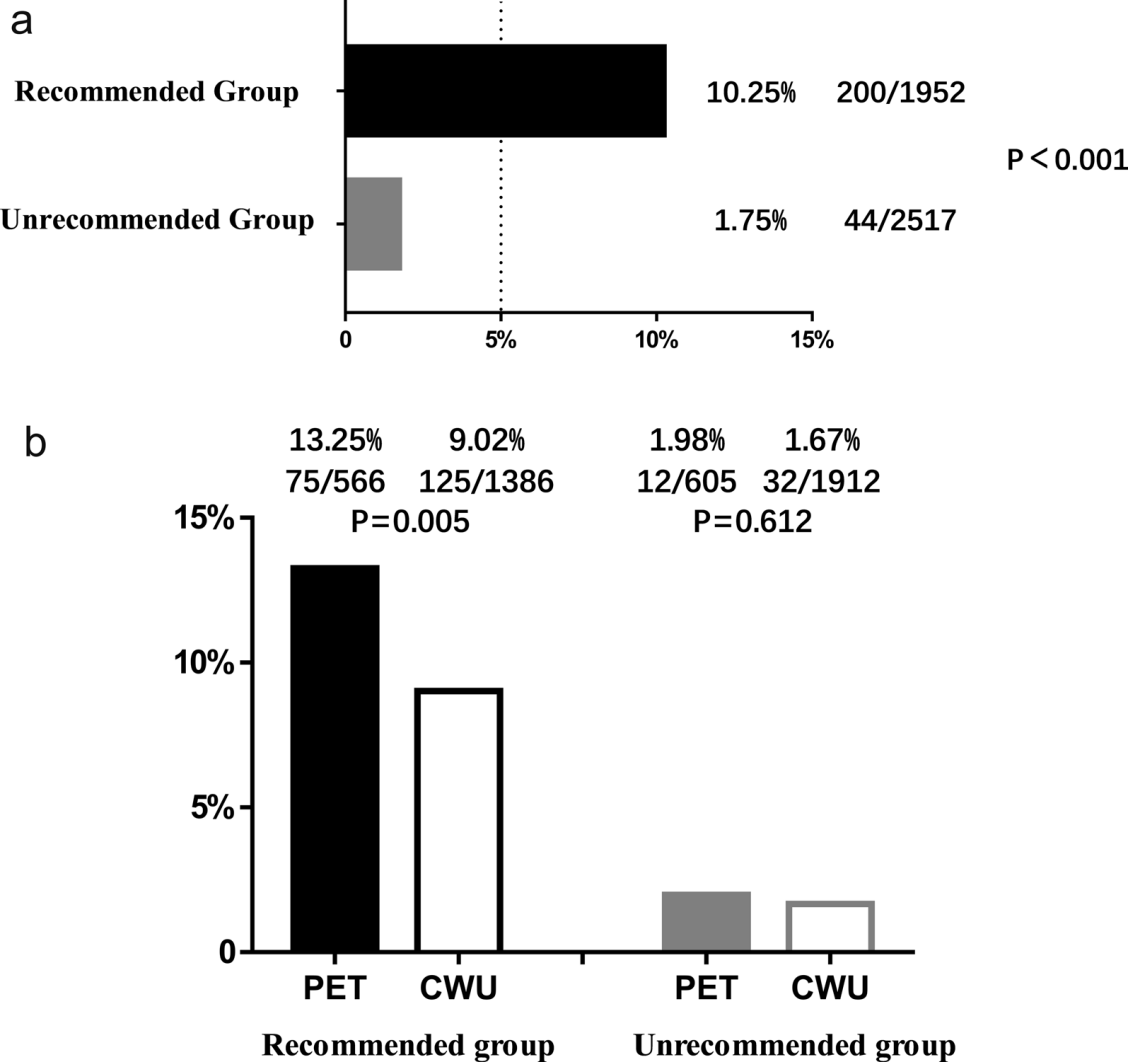
**a** Distant metastasis incidence in recommended group versus unrecommended group. **b** Distant metastasis incidence of PET/CT group versus CWU group in recommended patients and unrecommended patients, respectively

**Fig. 4:**
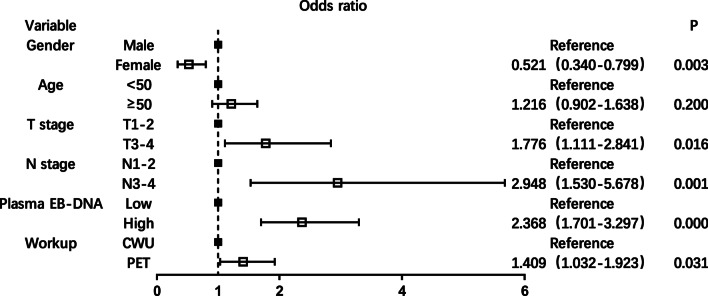
Multiple logistic regression analysis of the risk factors related to distant metastasis in the recommended group of de novo NPC patients

Based on the four risk factors identified in multivariate analysis, 16 situations were categorized based on combinations of these risk factors and the distant metastasis rates were calculated (Fig. 2). Patients with more risk factors showed a higher incidence of distant metastasis. The incidence of distant metastasis was 19.78% in subgroup of T3-4 stage, N2-3 stage, and male patients with high EBV DNA level, while it was merely 0.98% in subgroup of T1-2 stage, N0-1 stage, and female patients with low EBV DNA level. In probability theory, generally the small probability events close to zero (below 5%) is called a small probability event, it is essentially impossible to distinguish events [21]. According to the risk factors and incidence of distant metastasis, we subdivided all patients into two subgroups: patients with distant metastasis incidence above 5% were defined as recommended group and patients with distant metastasis incidence below 5% were defined as unrecommended group. Distant metastasis incidence was significantly different between the two groups (10.25% vs 1.75%, *P* < 0.001; Fig. 3a). The recommended group include: T3-4 stage and N2-3 stage patients regardless of the sex and high EBV DNA level; N0-1 stage and male patients with high EBV DNA level regardless of the T stage; T1-2 stage, N2-3 stage, and high EBV DNA patients regardless of the sex. In the recommended group, PET/CT significantly improved the detection rate of distant metastasis (13.25% vs 9.02%, *P* = 0.005; Fig. 3b). On multiple logistic regression, in the recommended group, PET (OR = 1.409, 95% CI 1.032–1.923) showed a significant correlation with distant metastasis (Fig. 4). To summarize, the following subset of patients with de novo NPC were found to particularly benefit from PET/CT for detection of distant metastases: T3-4 stage combined with N2-3 stage, high EBV DNA level combined with male sex, and N2-3 stage combined with high EBV DNA level.

### Cost-effectiveness analysis

In Fujian province, China, the cost of whole-body ^18^F-FDG PET/CT and CWU is $ 1202.06 (8400-yuan RMB) and $ 238.23 (1664.73-yuan RMB), respectively. In the entire cohort, the ICER was $ 36,098.50 (252,258.05-yuan RMB) and at the WTP threshold of $32,700.00 (228,507.6-yuan RMB), PET/CT is not cost-effective. Comfortingly, in the recommended group, the ICER was $22,785.58 (159,043.34-yuan RMB), which implies that additional cost for every one percent increase in distant metastasis detection rate was $22,785.58 by PET/CT. However, in the unrecommended group, the ICER for PET/CT was $310,912.90 (2,172,674.19-yuan RMB).

## Discussion

The present study was a retrospective analysis of a large cohort of real-world patients. We found that PET/CT significantly improved the detection rate of distant metastasis in de novo NPC patients wtih high-risk factors compared to CWU. Despite the high cost, PET/CT was found to be cost-effective for high-risk patients in the recommended group.

Due to its unique anatomical position and tendency for occult dissemination, NPC is associated with one of the highest incidences of distant metastasis among all head-and-neck squamous cell carcinomas [13]. In the present study, the incidence of distant metastasis in a large cohort in endemic areas was 5.46%. The previous studies demonstrated that the superior diagnostic sensitivity of PET/CT compared to CWU. However, more than 70% new NPC cases occur in developing countries with low per capita incomes and poor health infrastructure. In such settings, it is not cost effective to use PET/CT to screen NPC cases to identify metastasis. The European Society of Medical Oncology (ESMO) briefly recommended PET/CT for locally advanced NPC in its latest ESMO-EURACAN clinical practice guidelines [22]. However, the guidelines do not adequately identify the population at risk, since patients with locally advanced NPC account for more than 70% of newly diagnosed cases. Therefore, identification of clinical risk factors for distant metastasis is a key imperative to selectively recommend PET/CT to patients with relatively higher tendency for metastasis.

In most previous studies about distant metastasis of NPC, N stage and EBV DNA level were found to be significant risk factors. Higher N stage and plasma EBV DNA level were independent predictors of distant metastasis and poor survival outcomes [17, 23–25]. However, there is no clear consensus on the correlation of T stage, sex, and age with distant metastasis. Some studies suggested that these are not risk factors for distant metastasis. On the contrary, in the study by Xie et al. [26], T stage, age-group, and EBV level were found to be independent risk factors for distant metastasis. In a propensity-matched analysis of 5929 Chinese patients with NPC, sex was found to significantly affect distant metastasis-free survival and females showed significant advantage over males in this respect [27]. It is a reasonable strategy to screen for these risk factors and increase the diagnosis efficiency. In our study, sex, T stage, N stage, and pretreatment plasma EBV DNA level showed a strong association with distant metastasis. In multivariate analysis, male sex, T3-4 stage, N2-3 stage, and high plasma EBV DNA (≥ 14,650 copies/mL) were independent predictors of distant metastasis. Based on the findings, the following three combinations of risk factors showed a greater propensity for metastases: T3-4 stage combined with N2-3 stage, high EBV DNA level combined with male sex, and N2-3 stage combined with high EBV DNA level. Thus, patients with these combinations of risk factors seem suitable candidates for PET/CT to allow for precise M staging. In high-risk patients of the recommended group, the detection rate of distant metastasis with PET/CT (13.25%) was significantly higher than that with CWU (9.02%).

The reported overall survival period for patients with de novo metastatic NPC varies from months to years [28]. Thus further studies are required to investigate the optimal treatment pattern in patients with de novo metastatic NPC. Based on the available data from clinical studies, the committee of NPC of the Chinese Anti-Cancer Association (CACA) reached a preliminary consensus on the treatment strategy for metastatic NPC [6]. In a phase 3 randomized clinical trial, You et al. reported the first level 1 evidence to support the use of sequential locoregional radiotherapy for patients with metastatic NPC; the results helped improve the standard treatment for metastatic NPC [29, 30]. Early diagnosis of metastasis can help prolong the life span by up to a few years in some patients. Expensive test with high-end equipment can help identify metastasis patients at a early stage.

Bearing in mind that health care costs are a key determinant of clinical decision-making, it is crucial to strike the best balance between costs and effectiveness. The diagnostic value of PET/CT has been verified in the context of NPC, cancer of unknown primary in head and neck, non-small cell lung cancer, and other tumors [31–33]. However, PET is not universally available because of the high capital and operating costs. Thus, it is important to identify patients with a relatively higher tendency for metastasis who are more likely to benefit from PET/CT. In the present study, we merely considered the detection rates of distant metastasis as a measure of effectiveness to determine the ICER. At a WTP threshold of $32,700.00 (228,507.6-yuan RMB), the ICER for the whole cohort was $ 36,098.50 (252,258.05-yuan RMB), which means that PET/CT was not cost-effective compared to CWU within the patient population as a whole. However, based on the high-risk factors, the ICER in the recommended group was $22,785.58 (159,043.34-yuan RMB), and $310,912.90 (2,172,674.19-yuan RMB) in the unrecommended group. The additional cost for every one percent increase in distant metastasis detection rate was mere $22,785.58 in recommended patients. Thus, use of PET/CT seems to be cost-effective in de novo NPC patients who have a higher tendency for metastasis in endemic areas.

Some limitations of our study should be considered while interpreting the results. First, as a retrospective research, we could not conduct cross validation between PET/CT group and CWU group; in addition, metastases occurring during treatment were not considered in the analysis. Thus, we could not identify false-positive results and only assessed the pre-treatment status. Second, due to the inherent limitations of a retrospective study, our cost-effectiveness analysis did not include a decision tree analysis and estimation of quality-adjusted life years (QALY). Further prospective trials are required to provide more robust evidence of the diagnostic value of PET in patients with NPC.

## Conclusion

Despite its effectiveness, wide application of PET/CT at the time of initial diagnosis may not be cost-effective. Compared to conventional work-up, PET/CT significantly improves the detection rates of distant metastases in the subset of NPC patients at high-risk in endemic areas. To be specific, PET is a cost-effective strategy for patients with the following combinations of high-risk factors: T3-4 stage combined with N2-3 stage, high EBV DNA (≥ 14,650 copies/mL) combined with male sex, and N2-3 stage combined with high EBV DNA level.

## Data Availability

Data are available upon reasonable request. The data sets generated during and/or analyzed during the current study are available from the corresponding author on reasonable request.

## Abbreviations

AJCC: American Joint Committee on Cancer
AUC: Area under curve
CACA: Chinese Anti-Cancer Association
CIs: Confidence intervals
CNY: China Yuan
CT: Computed tomography
CWU: Conventional work-up
EBV: Epstein-Barr virus
ESMO: European Society of Medical Oncology
^18^F-FDG PET/CT: [^18^F] fluorodeoxyglucose positron emission tomography and computed tomography
GDP: Gross Domestic Product
ICER: Incremental cost-effectiveness ratio
MRI: Magnetic resonance imaging
NPC: Nasopharyngeal carcinoma
ORs: Odds ratios
QALY: Quality-adjusted life years
q-PCR: Quantitative polymerase chain reaction
ROC: Receiver operating characteristic
USD: U.S. Dollars
WTP: Willingness-to-pay

## References

[1] Chen YP, Chan ATC, Le QT, Blanchard P, Sun Y, Ma J (2019). Nasopharyngeal carcinoma. Lancet.

[2] Sung H, Ferlay J, Siegel RL, Laversanne M, Soerjomataram I, Jemal A (2021). Global cancer statistics 2020: GLOBOCAN estimates of incidence and mortality worldwide for 36 cancers in 185 countries. CA Cancer J Clin.

[3] Liu F, Xiao JP, Xu GZ, Gao L, Xu YJ, Zhang Y (2013). Fractionated stereotactic radiotherapy for 136 patients with locally residual nasopharyngeal carcinoma. Radiat Oncol.

[4] Chua MLK, Wee JTS, Hui EP, Chan ATC (2016). Nasopharyngeal carcinoma. Lancet.

[5] Lee AWM, Ng WT, Chan LK, Chan OSH, Hung WM, Chan CC (2012). The strength/weakness of the AJCC/UICC staging system (7th edition) for nasopharyngeal cancer and suggestions for future improvement. Oral Oncol.

[6] Chen XZ, Li JG, Lin SJ, Hu CS (2018). Expert consensus on the treatment of metastasis nasopharyngeal carcinoma. Chin J Radiat Oncal.

[7] GLOBOCAN cancer statistics. https://globocan.iarc.fr/Pages/fact_sheetspopulation.aspx. Accessed 2 Jan 2017.

[8] Xu C, Zhang Y, Peng L, Liu X, Li WF, Sun Y (2017). Optimal modality for detecting distant metastasis in primary nasopharyngeal carcinoma during initial staging: a systemic review and meta-analysis of 1774 patients. J Cancer.

[9] Liu FY, Lin CY, Chang JT, Ng SH, Chin SC, Wang HM (2007). 18F-FDG PET can replace conventional work-up in primary M staging of nonkeratinizing nasopharyngeal carcinoma. J Nucl Med.

[10] Buck AK, Herrmann K, Stargardt T, Dechow T, Krause BJ, Schreyögg J (2010). Economic evaluation of PET and PET/CT in oncology: evidence and methodologic approaches. J Nucl Med.

[11] Jerusalem G, Hustinx R, Beguin Y, Fillet G (2002). The value of positron emission tomography (PET) imaging in disease staging and therapy assessment. Ann Oncol.

[12] Vellayappan BA, Soon YY, Earnest A, Zhang Q, Koh WY, Tham IW (2014). Accuracy of (18)F-flurodeoxyglucose-positron emission tomography/computed tomography in the staging of newly diagnosed nasopharyngeal carcinoma: a systematic review and meta-analysis. Radiol Oncol.

[13] Chang JT, Chan SC, Yen TC, Liao CT, Lin CY, Lin KJ (2005). Nasopharyngeal carcinoma staging by (18)F-fluorodeoxyglucose positron emission tomography. Int J Radiat Oncol Biol Phys.

[14] Zhou H, Shen G, Zhang W, Cai H, Zhou Y, Li L (2016). 18F-FDG PET/CT for the diagnosis of residual or recurrent nasopharyngeal carcinoma after radiotherapy: a metaanalysis. J Nucl Med.

[15] Fei Z, Chen C, Huang Y, Qiu X, Li Y, Li L (2019). Metabolic tumor volume and conformal radiotherapy based on prognostic PET/CT for treatment of nasopharyngeal carcinoma. Medicine (Baltimore).

[16] Zhang L, Dong D, Li H, Tian J, Ouyang F, Mo X (2019). Development and validation of a magnetic resonance imaging-based model for the prediction of distant metastasis before initial treatment of nasopharyngeal carcinoma: a retrospective cohort study. EBioMedicine.

[17] Ren YY, Li YC, Wu HB, Wang QS, Han YJ, Zhou WL (2017). Whole-body 18F-FDG PET/CT for M staging in the patient with newly diagnosed nasopharyngeal carcinoma: who needs?. Eur J Radiol.

[18] Hojjat H, Svider PF, Folbe AJ, Raza SN, Carron MA, Shkoukani MA (2017). Cost-effectiveness of routine computed tomography in the evaluation of idiopathic unilateral vocal fold paralysis. Laryngoscope.

[19] Eichler HG, Kong SX, Gerth WC, Mavros P, Jönsson B (2004). Use of cost-effectiveness analysis in health-care resource allocation decision-making: how are cost-effectiveness thresholds expected to emerge?. Value Health.

[20] Chen Z, Zhan M, Tian F, Xu T (2020). Cost-effectiveness analysis of the addition of bevacizumab to temozolomide therapy for the treatment of unresected glioblastoma. Oncol Lett.

[21] Dallal GE (2012). The little handbook of statistical practice.

[22] Bossi P, Chan AT, Licitra L, Trama A, Orlandi E, Hui EP (2020). Nasopharyngeal carcinoma: ESMO-EURACAN Clinical Practice Guidelines for diagnosis, treatment and follow-up. Ann Oncol.

[23] Tang LQ, Chen QY, Fan W, Liu H, Zhang L, Guo L (2013). Prospective study of tailoring whole-body dual-modality [18F] fluorodeoxyglucose positron emission tomography/computed tomography with plasma Epstein-Barr virus DNA for detecting distant metastasis in endemic nasopharyngeal carcinoma at initial staging. J Clin Oncol.

[24] Liu FY, Chang JT, Wang HM, Liao CT, Kang CJ, Ng SH (2006). [18F] fluorodeoxyglucose positron emission tomography is more sensitive than skeletal scintigraphy for detecting bone metastasis in endemic nasopharyngeal carcinoma at initial staging. J Clin Oncol.

[25] Ai QY, King AD, Mo FKF, Law BKH, Bhatia KS, Ma BB (2017). Prediction of distant metastases from nasopharyngeal carcinoma: Improved diagnostic performance of MRI using nodal volume in N1 and N2 stage disease. Oral Oncol.

[26] Xie C, Li H, Yan Y, Liang S, Li Y, Liu L (2020). A nomogram for predicting distant metastasis using nodal-related features among patients with nasopharyngeal carcinoma. Front Oncol.

[27] OuYang PY, Zhang LN, Lan XW, Xie C, Zhang WW, Wang QX (2015). The significant survival advantage of female sex in nasopharyngeal carcinoma: a propensity-matched analysis. Br J Cancer.

[28] Sun XS, Liang YJ, Chen QY, Guo SS, Liu LT, Sun R (2020). Optimizing the treatment pattern for de novo metastatic nasopharyngeal carcinoma patients: a large-scale retrospective cohort study. Front Oncol.

[29] You R, Liu YP, Huang PY, Zou X, Sun R, He YX (2020). Efficacy and safety of locoregional radiotherapy with chemotherapy vs chemotherapy alone in de novo metastatic nasopharyngeal carcinoma: a multicenter phase 3 randomized clinical trial. JAMA Oncol.

[30] Smith SM, Wachter K, Burris HA, Schilsky RL, George DJ, Peterson DE (2021). Clinical cancer advances 2021: ASCO's Report on progress against cancer. J Clin Oncol.

[31] Jerusalem G, Hustinx R, Beguin Y, Fillet G (2003). PET scan imaging in oncology. Eur J Cancer.

[32] Antoch G, Vogt FM, Freudenberg LS, Nazaradeh F, Goehde SC, Barkhausen J (2003). Whole-body dual-modality PET/CT and whole-body MRI for tumor staging in oncology. JAMA.

[33] Smith KA, Dort JC, Hall SF, Rudmik L (2015). Cost-effectiveness of positron emission tomography-CT in the evaluation of cancer of unknown primary of the head and neck. Head Neck.

